# Improving Pediatric Basic Life Support Performance Through Blended Learning With Web-Based Virtual Patients: Randomized Controlled Trial

**DOI:** 10.2196/jmir.4141

**Published:** 2015-07-02

**Authors:** Ronny Lehmann, Christiane Thiessen, Barbara Frick, Hans Martin Bosse, Christoph Nikendei, Georg Friedrich Hoffmann, Burkhard Tönshoff, Sören Huwendiek

**Affiliations:** ^1^ Center for Pediatrics and Adolescent Medicine Department of General Pediatrics University Hospital Heidelberg Heidelberg Germany; ^2^ Center for Pediatrics and Adolescent Medicine Department of General Pediatrics University Hospital Düsseldorf Düsseldorf Germany; ^3^ Department of General Internal Medicine and Psychosomatics University Hospital Heidelberg Heidelberg Germany; ^4^ Institute of Medical Education Department of Assessment and Evaluation Faculty of Medicine, University of Bern Bern Switzerland

**Keywords:** virtual patients, blended learning, simulation, pediatric basic life support, performance

## Abstract

**Background:**

E-learning and blended learning approaches gain more and more popularity in emergency medicine curricula. So far, little data is available on the impact of such approaches on procedural learning and skill acquisition and their comparison with traditional approaches.

**Objective:**

This study investigated the impact of a blended learning approach, including Web-based virtual patients (VPs) and standard pediatric basic life support (PBLS) training, on procedural knowledge, objective performance, and self-assessment.

**Methods:**

A total of 57 medical students were randomly assigned to an intervention group (n=30) and a control group (n=27). Both groups received paper handouts in preparation of simulation-based PBLS training. The intervention group additionally completed two Web-based VPs with embedded video clips. Measurements were taken at randomization (t0), after the preparation period (t1), and after hands-on training (t2). Clinical decision-making skills and procedural knowledge were assessed at t0 and t1. PBLS performance was scored regarding adherence to the correct algorithm, conformance to temporal demands, and the quality of procedural steps at t1 and t2. Participants’ self-assessments were recorded in all three measurements.

**Results:**

Procedural knowledge of the intervention group was significantly superior to that of the control group at t1. At t2, the intervention group showed significantly better adherence to the algorithm and temporal demands, and better procedural quality of PBLS in objective measures than did the control group. These aspects differed between the groups even at t1 (after VPs, prior to practical training). Self-assessments differed significantly only at t1 in favor of the intervention group.

**Conclusions:**

Training with VPs combined with hands-on training improves PBLS performance as judged by objective measures.

## Introduction

Basic life support training, such as for pediatric basic life support (PBLS), is usually simulation-based with the need for evaluating learners’ performances [1-4]. Although there is evidence that simulator training is effective to improve basic life support performance, literature comparing various methods of training is scarce [5]. In particular, the instructional design of life support training is increasingly being investigated. Carrero et al assessed the improvement in procedural knowledge acquired by typically used tutor-led, case-based discussions versus the use of noninteractive multimedia presentations—video plus PowerPoint presentation. Both were shown to have equal impact on the level of cognitive skills [6]. Some reports have shown advantages for learning basic life support when using instructional videos [7-9]. Such approaches provide individual preparation and can be easily distributed, save instructors’ resources, and allow for more training time in face-to-face sessions.

For promoting clinical reasoning and decision making, virtual patients (VPs) are known for being effective [10]. For the context of acquiring life support skills, VPs integrate features that have been shown to foster both the development of clinical decision making (eg, through interactivity and feedback [11]) and procedural skills (eg, by integration of media [12]). E-learning and blended learning approaches are gaining popularity in emergency medicine curricula [13-16]. Lehmann et al reported recently that VPs combined with skills laboratory training are perceived by both trainees and trainers as an effective approach to train undergraduates in PBLS, leading to an efficient use of training time [17]. A few other reports have already suggested positive effects of VPs and comparable simulators regarding knowledge and procedural skill acquisition used for different kinds of life support courses [18-20].

In this study, we investigated the effect of VPs combined with standard simulation-based PBLS training on the acquisition of clinical decision-making skills and procedural knowledge, objective skill performance, and self-assessment. Our hypotheses were that preparation with VPs would yield (1) superior clinical decision making and procedural knowledge, (2) an objectively better performance of PBLS after the training, and (3) better self-assessment after working with VPs and after exposure to standard training.

## Methods

### Study Design

**Figure 1:**
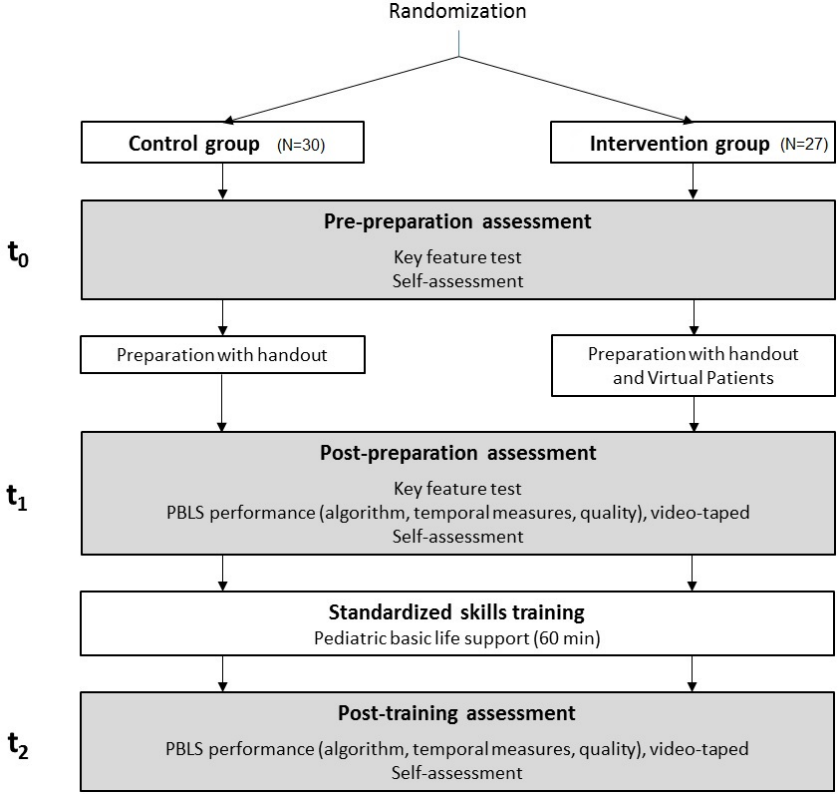
Study design.

We used a two-group randomized trial design (see Figure 1). All participants were assessed regarding their self-assessment, clinical decision-making skills, and procedural knowledge (key-feature test) about PBLS after randomization to ensure comparability (prepreparation assessment, t_0_). PBLS training sessions were conducted 1 to 2 weeks after the preparation assessment. Both groups were requested to prepare themselves a day ahead of the appointed training using handouts we had distributed. In addition, the intervention group (IG) was granted access to VPs as mandatory preparation. After the preparation, on the day of the practical training, self-assessment and procedural knowledge were assessed again to compare the participants’ progress (postpreparation assessment, t_1_). Subsequently, we videotaped PBLS sequences undertaken by each participant for later scoring of their performances. Both groups then attended standard training on PBLS. Later that day, we again recorded PBLS demonstrations and reevaluated participants’ self-assessments after the practical training (posttraining assessment, t_2_). The study was conducted in September 2014.

### Instruments

#### Overview

All instruments were pilot-tested on video recordings of PBLS demonstrations by student tutors and faculty before implementation, and revisions were made to ensure clarity and content validity. We particularly tested the estimated and calculated temporal scores adapted from international recommendations [21] by recording and analyzing best-practice examples of our faculty.

#### Basic Data

Participants were asked about their age, sex, and level of qualification in emergency medicine. For subgroup analysis we identified participants who were qualified as paramedics or had some similar training—qualifications that include PBLS training.

#### Clinical Decision-Making Skills and Procedural Knowledge

We developed a key-feature test according to published guidelines [22] to evaluate the students’ procedural knowledge and clinical decision making. This kind of testing was introduced by Page and Bordage specifically to assess clinical decision-making skills [23]. The test contained seven cases with three key features each (see Multimedia Appendix 1). Answers were to be given in “write in” format, which was suggested for decisions regarding the differential diagnosis, therapy, and further management [22]. Questions concerned both clinical decision making (proposed next steps) and procedural knowledge (eg, head positioning or compression depth). Each correct answer was given 1 point, with a maximum of 21 points. The test was reviewed for correctness and clinical relevance by group-blinded senior pediatricians with expertise in PBLS.

#### Performance: Adherence to Algorithm

Two raters scored the performed algorithm for its correct order. Each step of the sequence was given 2 points if it was done in the correct algorithmic order. It was given 1 point if it had been performed in an incorrect algorithmic order. No points were assigned if the step had not been undertaken at all (see Multimedia Appendix 2). The maximum score was 18.

#### Performance: Temporal Demands

Concrete temporal recommendations for three procedural steps of the PBLS algorithm are as follows [21].

1. Every rescue breath should take 1.0 to 1.5 s for inspiration plus time for expiration.

2. Assessment of the signs of life and circulation may not take longer than 10 s.

3. Chest compressions should be given at a frequency of at least 100/min, not exceeding 120/min.

With these recommendations being followed, the optimal temporal specifications for the initial five rescue breaths, the circulation check, and the four cardiopulmonary resuscitation (CPR) cycles were estimated and calculated (see Multimedia Appendix 3). The optimal total time was also estimated for the whole sequence, from safety check to emergency call. We scored 2 points for each procedural step if it was performed within ±10% of the optimal estimated calculated time and 1 point if within ±20%. If the participant took a longer or shorter time, no points were scored per step. Two raters measured these times on video recordings. A total of 8 points could be achieved.

#### Performance: Procedural Quality

Two group-blinded video raters with expertise in PBLS scored the procedural quality of the participants’ PBLS skills. The scores were averaged for further analysis. We used a scoring form in trichotomous fashion, with 2 points for correct performance, 1 point for minor deficits, and no points for major deficits (see Multimedia Appendix 4). A maximum of 22 points could be achieved; items were not weighted. Such kinds of scoring systems with comparable checklists are established to assess clinical performances in simulated emergency scenarios [24-27]. In contrast to published rating modalities, we rated the aspects of the algorithm and time measures separately as described above to achieve more objective scoring. In addition, skills performance levels were rated globally: competent, borderline, not competent. Only the performances that were rated “competent” concordantly by both raters were counted and used in the analyses.

#### Self-Assessments

We developed a self-assessment instrument consisting of seven items on procedural knowledge and seven items on procedural skills (see Multimedia Appendix 5). Two senior pediatricians with expertise in both PBLS and questionnaire design had reviewed these items. Answers were given on 100 mm visual analog scales from 0 (very little confidence) to 100 (highly confident).

### Preparation Material and Pediatric Basic Life Support Training

**Figure 2:**
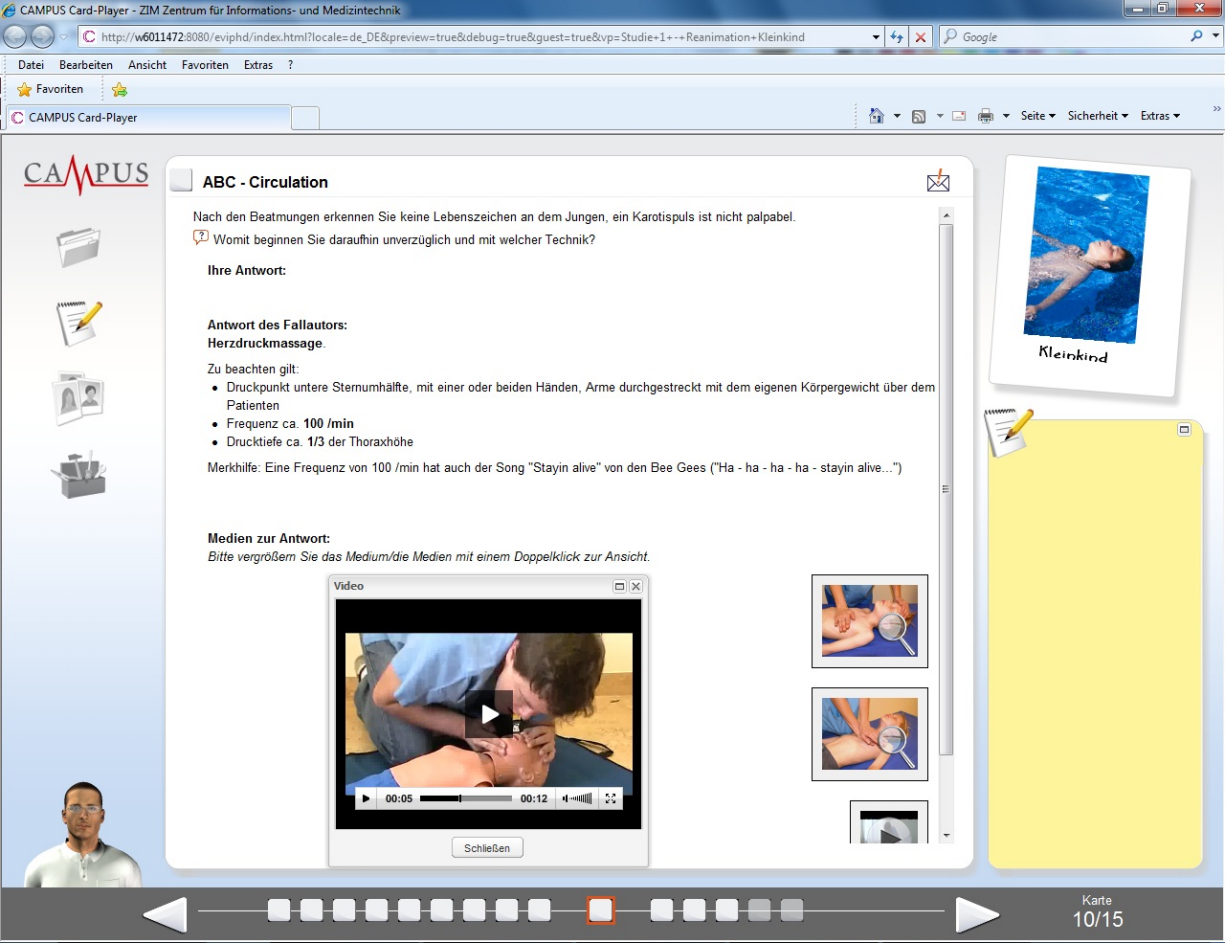
Screenshot of CAMPUS-Software showing a virtual patient.

For individual preparation of the training, we developed and distributed to both groups a paper handout on PBLS. Such handouts are commonly used as preparation for undergraduate skills laboratories [28]. The handout contained all relevant information, explaining the procedural steps of PBLS, including the algorithm, temporal demands, and a flowchart. Additionally, the intervention group was given Web-based access to two VPs dealing with PBLS in infants and toddlers. The VPs were designed with CAMPUS-Software [29] according to published design criteria [11] and enriched by video clips and interactive graphics (see Figure 2). For more detailed characterization of the VP cases used for this study, see Lehmann et al (VP3 and VP4) [17]. Both VPs had to be worked up twice, which was checked electronically but without the ability to identify any participant. The required overall workup time was estimated at 30 to 60 min based on previously measured log data.

Participants were trained in a single-rescuer scenario: from finding an unresponsive child, to the emergency call after 1 min (about four cycles) of CPR according to current guidelines [21]. The hands-on training was divided into two sessions—infant and toddler phases—of 30 min each. The sessions were structured with a commonly used four-step approach [1,28,30-32]. Steps three and four—tutor guided by learner and demonstration by the learner, respectively—were performed once per session by each participant so there was a standardized and comparable amount of individual training time. Two senior tutors provided close feedback on the participants’ performance as suggested by Issenberg et al for effective learning during simulations [33]. We used manikins by Laerdal Medical GmbH, Puchheim, Germany ("Baby Anne") and Simulaids Inc, Saugerties, New York ("Kyle").

### Participants and Data Collection

The Ethics Committee of the Medical Faculty Heidelberg granted ethical approval for this study (EK No. S-282/2014). All collected data were pseudonymized. We affirmed with participants by written informed consent that their participation was voluntary, that they could not be identified from the collected data, and that no plausible harm could arise from participation in the study.

We offered participation in this study to a total of about 480 third- and fourth-year medical students at Heidelberg Medical School by group emails and bulletin boards. Invited students had already completed basic life support (BLS) training but had had no PBLS training yet. Announcements were worded as invitations to a special PBLS course and educational study without mentioning e-learning in particular. At an orientation meeting, prospective students enrolled themselves onto a numbered list, unaware of group allocation, which was randomly distributed by numbers.

### Rater Selection and Training

We selected and trained two raters to score videotaped performances with the help of best-practice videos of senior faculty. Rater training included reviewing the case content and objectives, and an introduction to the rating schemes. Videotaped examples with different levels of procedural quality were discussed for calibration of the intended use of the schemes. We chose a senior pediatric consultant and a pediatric intensive care nurse practitioner, each of whom was an experienced facilitator for pediatric emergency simulations. Raters were blinded to group classification of all video records.

### Data Analysis

Results are presented as the mean ± standard deviation per group and given as the percent of the maximum achievable scores. Data were checked for normal distribution using the Kolmogorov-Smirnov test. If a presumed normal distribution was accepted, statistical differences were evaluated using the unpaired *t* test for between-group comparisons and the paired *t* test for within-group comparisons. Otherwise, we used the Mann-Whitney U test for nonnormal distributions. We assumed that a group difference of 1 SD or more was a relevant effect size. For a group of 30 subjects, we estimated a 65% power to detect this effect, assuming a two-sided significance level of .05. The interrater reliability was estimated using the case 2 intraclass correlation coefficient (ICC2) measured on 100% of the sample size [34]. Global competence-level ratings were compared using Fisher’s exact test. As a higher level of qualification in PBLS appeared to be a possible confounder, we confirmed all statistics with exclusion of participants with PBLS qualifications who were identified from the basic data. We used SPSS Statistics version 21 (IBM Corporation, Armonk, NY, USA) for all statistical analyses and an alpha level of .05.

## Results

### Overview

**Figure 3:**
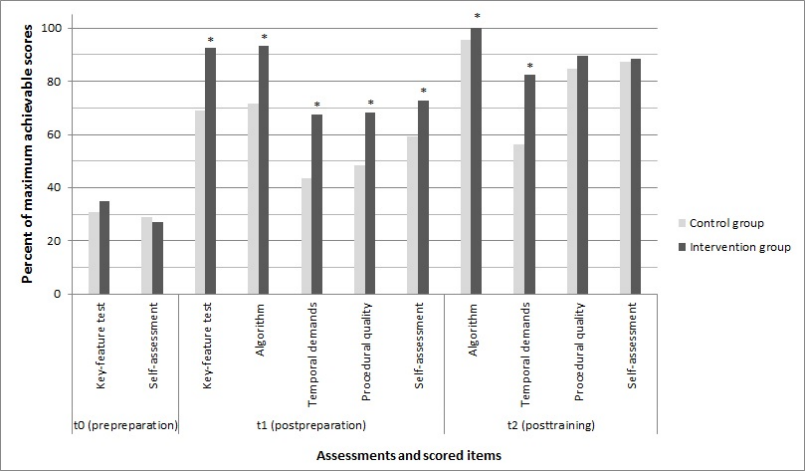
Key-feature test, performance, and self-assessment scores. Scores are given as the percent of the maximum achievable scores (*P<.05).

Scoring results are depicted in Table 1 and Figure 3.

**Table 1:** Key-feature test, performance, and self-assessment scores.

Scored items		Scores, mean (SD) or n (%)		*P* (CG vs IG)
		Control group (CG)	Intervention group (IG)	
**Procedural knowledge: Key-feature test (%),** **mean (SD)**				
	t_0_^a^	31.0 (12.9)	34.8 (17.1)	.34
	t_1_^b^	68.8 (16.3)	92.2 (4.7)	<*.001* ^c^
	*P* (t_0_ vs t_1_)	*<.001*	<*.001*	
**Performance: Adherence to algorithm (%),** **mean (SD)**				
	t_1_	72.0 (17.7)	93.4 (7.1)	<*.001*
	t_2_^d^	95.7 (7.2)	99.8 (1.1)	*.008*
	*P* (t_1_ vs t_2_)	<*.001*	<*.001*	
**Performance: Temporal demands (%),** **mean (SD)**				
	t_1_	43.3 (23.4)	67.6 (21.4)	<*.001*
	t_2_	55.8 (27.8)	82.4 (17.8)	<*.001*
	*P* (t_1_ vs t_2_)	*.03*	*.004*	
**Total time of PBLS** ^e^ **sequence in seconds, mean (SD)**				
	t_1_	107.7 (35.2)	88.1 (12.6)	*.008*
	t_2_	95.2 (16.2)	78.1 (10.2)	<*.001*
	*P* (t_1_ vs t_2_)	.05	<*.001*	
**Performance: Procedural quality (%), mean (SD)**				
	t_1_	48.8 (20.2)	68.2 (15.0)	<*.001*
	t_2_	84.4 (11.2)	89.4 (9.2)	.07
	*P* (t_1_ vs t_2_)	<*.001*	<*.001*	
**Global ratings (rated "competent"), n (%)**				
	t_1_	0/30 (0)	5/27 (19)	*.02*
	t_2_	17/30 (57)	23/27 (85)	*.02*
**Self-assessment (%), mean (SD)**				
	t_0_	29.1 (16.3)	27.2 (18.8)	.68
	t_1_	59.6 (15.8)	72.3 (11.7)	*.001*
	t_2_	87.4 (8.6)	88.4 (7.8)	.63

^a^Prepreparation assessment (t_0_).

^b^Postpreparation assessment (t_1_).

^c^Italicized *P* values represent significant results.

^d^Posttraining assessment (t_2_).

^e^Pediatric basic life support (PBLS).

### Basic Data

A total of 57 participants completed the training and all surveys were included in this study—30 (53%) in the control group (CG) and 27 (47%) in the intervention group; approximately 11.9% (57/480) of all eligible students. Out of 60 initial participants, 3 (5%) were excluded due to nonappearance at the training session; all participants of the intervention group processed the VPs completely as requested. Participants’ mean age was 24.2 years (SD 2.6) (16/30, 53% female) in the control group and 24.1 years (SD 3.1) (17/27, 63% female) in the intervention group. Of the 57 participants, there were 5 out of 30 (17%) PBLS-qualified participants (paramedics) in the control group and 4 out of 27 (15%) in the intervention group.

### Clinical Decision-Making Skills and Procedural Knowledge

There was no significant difference in the key-feature test results between the control group and intervention group at t_0_ (31.0%, SD 12.9 vs 34.8%, SD 17.1; *P*=.34). The intervention group showed a significantly superior increase in procedural knowledge at t_1_ compared with the control group (92.2%, SD 4.7 vs 68.8%, SD 16.3; *P*<.001). There were significant improvements in both groups between t_0_ and t_1_ (both *P*<.001).

### Performance: Adherence to Algorithm

Regarding adherence to the algorithm, the intervention group was already better than the control group at t_1_ (93.4%, SD 7.1 vs 72.0%, SD 17.7; *P*<.001), which continued at t_2_ (99.8%, SD 1.1 vs 95.7%, SD 7.2; *P*=.008). Significant improvements, however, were found between t_1_ and t_2_ for both groups (both *P*<.001).

### Performance: Temporal Demands

The intervention group already showed significantly better adherence to temporal specifications than the control group at t_1_ (67.6%, SD 21.4 vs 43.3%, SD 23.4; *P*<.001), which continued at t_2_ (82.4%, SD 17.8 vs 55.8%, SD 27.8; *P*<.001). Both groups showed significant improvements in temporal measures between t_1_ and t_2_ (*P*=.03 and *P*=.004, respectively). Table 1 also shows the measured mean times for the total sequence.

### Performance: Procedural Quality

The interrater reliability coefficient was .71 indicating a sufficient level of interrater agreement [35].

The performance quality score of the intervention group was significantly superior to that of the control group at t_1_ (68.2%, SD 15.0 vs 48.8%, SD 20.2; *P*<.001). After practical training, at t_2_, they did not differ significantly (89.4%, SD 9.2 vs 84.4%, SD 11.2; *P*=.07). Both groups showed significantly increased quality scores between t_1_ and t_2_ (both *P*<.001).

The global ratings of competence showed significant differences in favor of the intervention group, again already at t_1_ and continuing at t_2_ (0/30 CG vs 5/27 IG rated “competent”, *P*=.02; 17/30 CG vs 23/27 IG, *P*=.02, respectively). In all, 85% (23/27) of the intervention group participants performed PBLS “competently” after having practiced with VPs and undergoing PBLS training, compared with only 57% (17/30) of the control group participants.

### Self-Assessments

There was no significant difference in the self-assessment means of the two groups at t_0_ (29.1%, SD 16.3 CG vs 27.2%, SD 18.8 IG; *P*=.69). After different preparations, the intervention group showed a significant increase in its self-assessment compared with that in the control group at t_1_ (72.3%, SD 11.7 vs 59.6%, SD 15.8; *P*=.001). At t_2_, there was no significant difference between the groups (87.4%, SD 8.6 CG vs 88.4%, SD 7.8 IG; *P*=.62).

### Subgroup Analyses

After identifying and excluding PBLS-qualified participants, there were no changes in statistical significances in any of the calculations. The level of significance did not differ by the power of 10.

## Discussion

### Principal Findings

In this randomized controlled trial, we investigated the impact of an additional preparation with VPs on the improvement of objective and subjective learning outcomes of skill acquisition when combined with standard PBLS training. The control group and intervention group were comparable in terms of their self-assessment and procedural knowledge during the prepreparation assessment. However, after addition of practical training, the intervention group demonstrated significantly better performance in key aspects of PBLS than did the control group, although self-assessment ratings were similar. Also, after practicing with VPs, the intervention group had already demonstrated superior skills, even before the hands-on training in terms of objective skill performance, procedural knowledge, and self-assessment.

### Objective Learning Outcomes

After using VPs as an interactive preparation, the intervention group showed significantly improved clinical decision-making skills and procedural knowledge. Also, their PBLS skill performance was superior to that of the control group after the preparation period in regard to objective performance measures, including adherence to the algorithm, temporal demands, and procedural quality. This is in line with existing reports that such electronic learning activities improve both knowledge and skills [18,19]. De Vries et al also showed that a comparable computer simulator improves procedural skills [18]. Although they reported that some of the skill outcomes were suboptimal, the training was not blended with hands-on training as presented here, which led to increased improvements compared with using the computer simulator alone. Furthermore, as reported by Ventre et al, such approaches might fill a gap in continuing medical education [20]. Procedural skill performance was rated as objectively as possible to discriminate procedural learning effects. In contrast, typically used checklists often subsume adherence to the algorithm, temporal aspects, and performance quality—for example, “CPR continued—2 points for initiated immediately after pulse check and rhythm identification (<30 s) *and* good CPR technique *and* checks pulse with CPR” (taken from The Clinical Performance Tool [26]). At t_2_, when both groups had had equal practical training, the procedural steps of PBLS were still performed qualitatively more competently by the intervention group in some aspects. Such differences will probably not be found when using global rating scales, but may be when using automated skill reporting devices as used by Kononowicz et al [19], or when using discriminating rating schemes as presented here when such devices are not available.

We assume that VPs facilitated application of acquired clinical decision-making skills and procedural knowledge. Interactivity and feedback in VPs, which included interactive graphics and video clips, might have enhanced the learning process beyond the use of media, as in other approaches. It is well known that educational feedback, such as that given in the VPs, is the most important feature of simulation-based education [33]. Interacting with clinical case scenarios might also provide an emotionally activating stimulus to get trainees involved as it supports the acquisition and retention of skills [36]. For complex procedures, current learning theories support a reasonable simple-to-complex learning process that facilitates learning [37,38]. VPs may bridge this gap between knowledge and practice.

The presented results support the subjective perceptions of students and tutors [17] that such a blended learning approach is effective and efficient for procedural learning. In this study, self-directed learning with paper handouts seems to have had little effect on facilitating the acquisition of practical skills, although it did have an effect on improving procedural knowledge. In contrast, the blended approach that included interactive VPs for preparation led to improved learning of both procedural knowledge and procedural skills. Implications for CPR and other emergency training might be a more efficient and effective use of resource-intensive training time.

### Subjective Learning Outcomes

In their self-assessments, the participants of the intervention group judged themselves superior to those in the control group after the preparation period. Objective findings in their scored performances support these ratings. After their practical training, however, the self-assessments of the two groups were similar. In contrast, the intervention group still had superior objective scores regarding skill performance. Self-assessments are not necessarily correlated with performance; for example, postgraduate practitioners have limited ability to self-assess accurately, as shown by Davis et al [39].

### Study Strengths

The assessments of clinical decision-making skills and procedural knowledge, practical performance, and self-assessment combine relevant and detailed objective and subjective measures for elucidating the learning effects of this approach. This is one of the first studies that provides objective data that support how effectively VPs can foster the acquisition of PBLS skills.

### Study Limitations

Participants’ VP case completions were monitored to validate their workup, but the validation was not done in a controlled environment that allowed evaluation of participants’ efforts. Accordingly, efforts on the workup of handouts were also not assessed. Workup of VPs might have led to more motivation for learning even though the study was not announced as an e-learning study attracting mainly tech-savvy students. Because both groups had significantly increased procedural knowledge after the preparation period, we assumed that motivation for preparation might not have been very different. Result details, for example, of the temporal scoring, suggest that VPs address skills not affected by paper-based learning materials. However, group differences might also have been influenced by different durations of their efforts to learn in addition to different modalities. Additionally, both groups were not limited in their access to other learning resources than those provided. Also, most of the instruments used in this study have not been validated formally, although all were developed based on current literature and were pilot-tested. Finally, the sample size is rather limited, thereby providing limited power to investigate differences between groups.

### Conclusions

The blended learning approach described herein leads to improved outcomes of practical skill acquisition compared with a standard approach. Even before having practical training, preparation with VPs leads to improved practical performances as well as better clinical decision-making skills and procedural knowledge. Further studies are necessary to understand the specific benefit of using VPs regarding clinical skill acquisition and its sustainability.

## Appendix

Multimedia Appendix 1 Key-feature test.

Multimedia Appendix 2 Adherence to algorithm scoring form.

Multimedia Appendix 3 Temporal measures scoring form.

Multimedia Appendix 4 Performance quality scoring form.

Multimedia Appendix 5 Self-assessment instrument.

## Abbreviations

BLS: basic life support
CG: control group
CPR: cardiopulmonary resuscitation
ICC2: case 2 intraclass correlation coefficient
IG: intervention group
PBLS: pediatric basic life support
t0: prepreparation assessment
t1: postpreparation assessment
t2: posttraining assessment
VP: virtual patient
