# Supplementary material for: Improving Pediatric Basic Life Support Performance Through Blended Learning With Web-Based Virtual Patients: Randomized Controlled Trial
Source: J Med Internet Res. 2015 Jul 2;17(7):e162. doi: 10.2196/jmir.4141 (PMC4526972; doi:10.2196/jmir.4141)
Supplement: Multimedia Appendix 1 [file jmir_v17i7e162_app1.pdf]

Key-feature test (*correct answer*)

You suddenly locate a not moving 5-year old child on the water's edge of a lake. There is no one else around.<br>1. What is the most important thing to check first in this unknown situation?<br>*Safety (of rescuer and child).*<br>2. How do you assess the child's responsiveness?<br>*Call and stimulus.*<br>3. What do you do right next, when you assessed that the child is unresponsive?<br>*Shout for help.*
A 10-month old infant got unconscious and stopped breathing, after he accidently pulled a plastic bag over his head.<br>4. After starting ventilation, when are chest compressions indicated for this infant?<br>*Absent signs of life/circulation.*<br>5. If so, which frequency should be used for chest compressions on this infant?<br>*(At least) 100 per minute (not exceeding 120).*<br>6. Which ratio of chest compressions to ventilation should be used for CPR on this infant (CV ratio)?<br>*15 chest compressions : 2 rescue breaths.*
A 3-month old infant is suddenly found unresponsive lying in his bed. Someone else already went for calling the emergency hotline.<br>7. In which head position do you check if the infant is still breathing?<br>*Neutral position (and chin lift).*<br>8. What do you do right next after you assessed an apnea of this infant?<br>*5 (initial) rescue breaths.*<br>9. How do you do that in detail (which technique)?<br>*Mouth-to-mouth* or *mouth-to-nose/mouth.*
After a swimming accident of a 5-year old child you have to start ventilations and chest compressions.<br>10. Where is the optimal pressure point for chest compressions of this child?<br>*Lower half of the sternum.*<br>11. Which frequency should you use for chest compressions on this child?<br>*(At least) 100 per minute (not exceeding 120).*<br>12. How deep should the chest be compressed during chest compressions on this child?<br>*(At least) one-third of the depth of the chest.*
You just assessed that you have to ventilate a 10-month old infant which accidently fell into a swimming pool.<br>13. Which technique do you use to do so (without equipment)?<br>*Mouth-to-mouth or mouth-to-nose/mouth.*<br>14. What time should be used for the inspiration and expiration phase during external ventilation (in sec)?<br>*1s each.*<br>15. What do you do right after the initial rescue breaths on this infant?<br>*Assess signs of life/circulation.*
While spending the day on a lake you found an unresponsive 5-year old child floating in the water. You pulled him onto the beach and start basic life support.<br>16. Where can you try to check the pulse of this child?<br>*Carotid pulse in the neck (and femoral pulse).*<br>17. How long do you carry out basic life support until you would leave him to send an emergency call (if no one else did so yet)?<br>*1 minute* or *approximately 4 cycles of CPR 15:2.*<br>18. Besides the emergency call, would you (frequently) interrupt your CPR? If so, when and what for?<br>*No interruption.*
You just assessed that a 4-year old child is unresponsive to any stimulation.<br>19. What do you have to do to be able to assess his breath?<br>*Open airway* or *head tilt chin lift.*<br>20. After that, how do you assess the breath of this child?<br>*Look, listen and feel.*<br>21. When do you have to ventilate this child (concrete indication)?<br>*If not breathing normally.*
