# Supplementary material for: Improving Pediatric Basic Life Support Performance Through Blended Learning With Web-Based Virtual Patients: Randomized Controlled Trial
Source: J Med Internet Res. 2015 Jul 2;17(7):e162. doi: 10.2196/jmir.4141 (PMC4526972; doi:10.2196/jmir.4141)
Supplement: Multimedia Appendix 2 [file jmir_v17i7e162_app2.pdf]

Adherence to algorithm scoring

Task	2 points	1 point	0 points
1. Safe approach	done at correct algorithmic position	done but at wrong algorithmic position	not done
2. Asses responsiveness			
3. Shout for help			
4. Open airway			
5. Assess breathing			
6. Initial rescue breaths			
7. Assess signs of life/circulation			
8. (4) cycles of CPR			
9. Emergency call			
