# Supplementary material for: Improving Pediatric Basic Life Support Performance Through Blended Learning With Web-Based Virtual Patients: Randomized Controlled Trial
Source: J Med Internet Res. 2015 Jul 2;17(7):e162. doi: 10.2196/jmir.4141 (PMC4526972; doi:10.2196/jmir.4141)
Supplement: Multimedia Appendix 3 [file jmir_v17i7e162_app3.pdf]

Temporal measures scoring

Task	Calculated/estimated ideal	2 points	1 point	0 points
**Initial 5 rescue breaths**	5*(1 s inspiration + 1 s expiration)<br>≅<br>10 s	9–11 s<br><br>(± 10%)	8–9 s<br>*or*<br>11–12 s<br><br>(± 20%)	<8<br>*or*<br>>12 s
**Assess circulation**	---	<10 s	---	>10 s
**4 cycles CPR**<br>(excluding initial rescue breaths)	4*15 chest compressions at 100 per minute (36 s)<br>+<br>3*2 rescue breaths (12 s)<br>+<br>2 s for transitions<br>≅<br>50 s	45–55 s<br><br>(± 10%)	40–45 s<br>*or*<br>56–60 s<br><br>(± 20%)	<40 s<br>*or*<br>>60 s
**Total time of sequence** from approach to emergency call	Safe approach and responsiveness assessment, shout for help (≅ 10 s)<br>+<br>Initial 5 rescue breaths (10 s)<br>+<br>Circulation check (≅ 10 s)<br>+<br>4 cycles CPR (50 s)<br>≅<br>80 s	72–88 s<br><br>(± 10%)	64–71 s<br>*or*<br>89–96 s<br><br>(± 20%)	<64 s<br>*or*<br>>96 s
