# Supplementary material for: Improving Pediatric Basic Life Support Performance Through Blended Learning With Web-Based Virtual Patients: Randomized Controlled Trial
Source: J Med Internet Res. 2015 Jul 2;17(7):e162. doi: 10.2196/jmir.4141 (PMC4526972; doi:10.2196/jmir.4141)
Supplement: Multimedia Appendix 4 [file jmir_v17i7e162_app4.pdf]

Performance quality scoring

Task	2 points	1 point	0 points
**Safe approach**	Looked around for any danger	Done too flighty	Not done or not ratable
**Assess responsiveness**	Called loud *and* stimulated	Only called *or* stimulated	Not done or very poor
**Open airway**	**Infant:** head in neutral position, chin lift<br>**Toddler:** head tilt, chin lift	Partially done incorrectly	Not done or very poor
**Assess breathing**	**Look, listen and feel** (own cheek over patients‘ mouth, hand on patients’ upper abdomen or chest, looks downward to chest)	Does not assess *all three* dimensions	Not done or very poor
**Initial rescue breaths**	**Five sufficient** rescue breaths with visible chest rise and **correct head position**	Partially done insufficiently	Not done or very poor
**Assess circulation**	**Complete inspection** of signs of life (movements) and feels **carotid pulse**	Feels other pulse than carotid, does not look for movements	Not done or very poor
**CPR**	**CV ratio**<br>- 15 chest compressions in alteration with two rescue breaths	Partially done incorrectly	Not done or very poor
	**Chest compression procedure**<br>- Pressure point lower half of sternum<br>- Compression at least one-third of the depth of the chest<br>- Complete release after each compression	Partially done incorrectly	Not done or very poor
	**Chest compression technique**:<br>**Infant:** two or three fingers<br>**Toddler:** one or two hands	Partially done incorrectly	Not done or very poor
	**Rescue breaths**<br>Sufficient with visible chest rise and correct head position	Partially done insufficiently	Not done or very poor
	**Efficient transitions** between chest compressions and rescue breaths, e.g. positioning, fixed head position during chest compressions	Few interruptions during CPR	Many interruptions during CPR
