# Supplementary material for: Improving Pediatric Basic Life Support Performance Through Blended Learning With Web-Based Virtual Patients: Randomized Controlled Trial
Source: J Med Internet Res. 2015 Jul 2;17(7):e162. doi: 10.2196/jmir.4141 (PMC4526972; doi:10.2196/jmir.4141)
Supplement: Multimedia Appendix 5 [file jmir_v17i7e162_app5.pdf]

Self-assessment

**Knowledge**<br>I am confident to know…<br><br>1. how to assess responsiveness of toddlers and infants.<br>2. how to assess breathing of toddlers and infants.<br>3. how to open the airway of toddlers and infants according to the age with the correct technique.<br>4. how to ventilate a toddler or infant correctly without equipment.<br>5. when to start chest compressions on a toddler or infant.<br>6. how to carry out chest compressions on a toddler or infant.<br>7. the correct algorithm when locating an unresponsive toddler or infant according to current guidelines.
**Skills**<br>I possess confident practical skills to…<br><br>8. assess responsiveness of a toddler or infant.<br>9. assess breathing of a toddler or infant.<br>10. open the airway of a toddler or infant according to the age with the correct technique.<br>11. ventilate a toddler or infant correctly without equipment.<br>12. assess circulation of a toddler or infant.<br>13. how to carry out chest compressions on a toddler or infant.<br>14. apply the correct algorithm of current guidelines to a unresponsive toddler or infant.
Answers: on a 100 millimeter visual analogue scale from 0=very little confident to 100=highly confident
